# On origin of genetic code and tRNA before translation

**DOI:** 10.1186/1745-6150-6-14

**Published:** 2011-02-22

**Authors:** Andrei S Rodin, Eörs Szathmáry, Sergei N Rodin

**Affiliations:** 1 Human Genetics Center, School of Public Health, University of Texas, Houston, TX 77225, USA; 2 Collegium Budapest (Institute for Advanced Study), Szentháromság u. 2, H-1014 Budapest, Hungary; 3 Parmenides Center for the Study of Thinking, Kirchplatz 1, D-82049 Munich/Pullach, Germany; 4 Institute of Biology, Eötvös University, 1c Pázmány Péter sétány, H-1117 Budapest, Hungary; 5 Department of Molecular and Cellular Biology, Beckman Research Institute of the City of Hope, Duarte, CA 91010, USA

## Abstract

**Background:**

Synthesis of proteins is based on the genetic code - a nearly universal assignment of codons to amino acids (aas). A major challenge to the understanding of the origins of this assignment is the archetypal "key-lock vs. frozen accident" dilemma. Here we re-examine this dilemma in light of 1) the fundamental veto on "foresight evolution", 2) modular structures of tRNAs and aminoacyl-tRNA synthetases, and 3) the updated library of aa-binding sites in RNA aptamers successfully selected *in vitro *for eight amino acids.

**Results:**

The aa-binding sites of arginine, isoleucine and tyrosine contain both their cognate triplets, anticodons and codons. We have noticed that these cases might be associated with palindrome-dinucleotides. For example, one-base shift to the left brings arginine codons CGN, with CG at 1-2 positions, to the respective anticodons NCG, with CG at 2-3 positions. Formally, the concomitant presence of codons and anticodons is also expected in the reverse situation, with codons containing palindrome-dinucleotides at their 2-3 positions, and anticodons exhibiting them at 1-2 positions. A closer analysis reveals that, surprisingly, RNA binding sites for Arg, Ile and Tyr "prefer" (exactly as in the actual genetic code) the anticodon(2-3)/codon(1-2) tetramers to their anticodon(1-2)/codon(2-3) counterparts, despite the seemingly perfect symmetry of the latter. However, since *in vitro *selection of aa-specific RNA aptamers apparently had nothing to do with translation, this striking preference provides a new strong support to the notion of the genetic code emerging before translation, in response to catalytic (and possibly other) needs of ancient RNA life. Consistently with the pre-translation origin of the code, we propose here a new model of tRNA origin by the gradual, Fibonacci process-like, elongation of a tRNA molecule from a primordial coding triplet and 5'DCCA3' quadruplet (D is a base-determinator) to the eventual 76 base-long cloverleaf-shaped molecule.

**Conclusion:**

Taken together, our findings necessarily imply that primordial tRNAs, tRNA aminoacylating ribozymes, and (later) the translation machinery in general have been co-evolving to ''fit'' the (likely already defined) genetic code, rather than the opposite way around. Coding triplets in this primal pre-translational code were likely similar to the anticodons, with second and third nucleotides being more important than the less specific first one. Later, when the code was expanding in co-evolution with the translation apparatus, the importance of 2-3 nucleotides of coding triplets "transferred" to the 1-2 nucleotides of their complements, thus distinguishing anticodons from codons. This evolutionary primacy of anticodons in genetic coding makes the hypothesis of primal stereo-chemical affinity between amino acids and cognate triplets, the hypothesis of coding coenzyme handles for amino acids, the hypothesis of tRNA-like genomic 3' tags suggesting that tRNAs originated in replication, and the hypothesis of ancient ribozymes-mediated operational code of tRNA aminoacylation not mutually contradicting but rather co-existing in harmony.

**Reviewers:**

This article was reviewed by Eugene V. Koonin, Wentao Ma (nominated by Juergen Brosius) and Anthony Poole.

## Background

**Figure 1:**
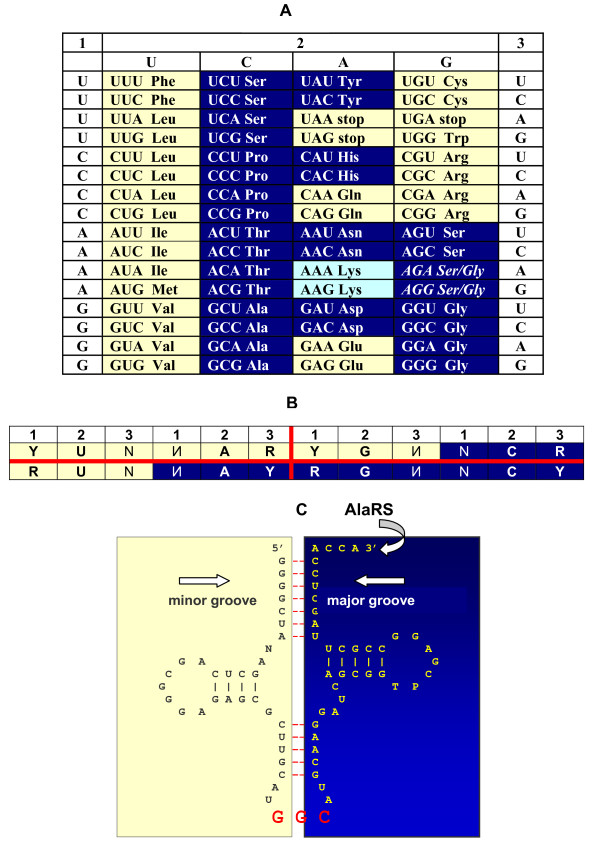
**The genetic code (adopted from **[19,24,36,40]). **A**. The code table. Yellow and blue colors indicate the two modes of tRNA recognition by aaRSs - from the minor and major groove sides of the acceptor stem, respectively [21,22]. The minor groove side of recognition represents mostly the class I aaRSs, the major groove side, class II aaRSs. Stop codons are shown in yellow because the known cases of their "capture" by amino acids are mostly from class I; AGG and AGA are assigned not to yellow Arg's codons (as they usually are) but to blue Ser's or Gly's codons (as they are in mitochondria) [68]. Lysine is colored in lighter shade of blue because some archaebacteria use class I synthetases for this amino acid [92]. **B**. The condensed version of the code representation when complementary codons are put *vis-á-vis *each other. This particular "yin-yang" version reveals otherwise invisible rules of the sub-code for two modes of tRNA aminoacylation described in [19,36]. These rules when applied to the pairs of tRNAs with complementary anticodons flanked by 5'U and R3' minimize a risk of their confusion by aaRSs. Symbols: N and complementary И denote all four nucleotides, R purine (G or A), Y pyrimidine (C or U). For details see [19,36]. **C**. The two-dimensional cloverleaf representation of a tRNA molecule (the *E. coli *tRNA^Ala ^with GGC anticodon is shown) [24]. The complementary halves are colored yellow (5' half) and blue (3' half), in accordance with the sub-code for two modes of tRNA aminoacylation (B). Arrows show the two sides from which the putative ribozymic precursors of class I and class II p-aaRSs recognized the proto-tRNAs.

Genetic Code refers to a nearly universal assignment of triplets (codons) of nucleotides (nts) to amino acids (aas), linking hereditary entities to the functional blocks of life (Figure 1A). In practice, this codon-to-aa assignment is realized through the agencies of 1) the code adaptor molecules of transfer RNAs (tRNAs) with a codon's complementary replica (anticodon) and the corresponding aa attached to the 3' end, and 2) aminoacyl tRNA synthetases (aaRSs), the enzymes that actually recognize and connect proper aa and tRNAs (Figure 1B and 1C).

Unraveling the origin of the genetic code and translation machinery is an inherently difficult problem [1]. On one hand, the system that unifies the nt and aa languages must necessarily be highly optimized. On the other hand, the events shaping the genetic code took place long time ago, and due to the relative compactness of the extant genetic code, many alternative scenarios for its origin are conceivable (and have been put forward).

Furthermore, the very existence of the two languages (with the code being a translational intermediary) implies an evolutionary motivation. This is a great challenge to evolutionists, even at the most abstract conceptual level of reasoning. When seeking, for example, the arguments for perhaps the most fateful transition in life history - from ribozymes to enzymes [2] - we cannot simply fall back on the truism that proteins are more efficient and versatile catalysts than RNAs. Indeed, proteins constitute a final product in the long chain of reactions performed by the very complex coding and translation machinery. The very complexity of the translation system inevitably suggests it has been shaped gradually ([3] see also [4-7]). However, the natural selection works strictly "in the present moment", right here and right now, just like a first-aid ambulance [8] - lacking the foresight of potential future advantages [2,7]. By contrast, any advantage of proteins over ribozymes could have materialized only in the very end of the ribozymes-mediated multi-step coding and translation processes. Therefore, just as with any case of step-by-step evolution towards a more complex system, there should be an evolutionary rationale behind each intermediate step. As far as the coding and translation system is concerned, it would seem logical to start with separating the two "origins" - origin of the code, and origin of translation [9-11]. Moreover, because life never evolves with foresight, we inevitably arrive to the hypothesis that the code emerged before translation - in response to the needs of the RNA world (ibid).

The detailed treatment of the pre-translational origin of the genetic code is a whole separate topic that will be reviewed elsewhere (Szathmáry and Rodin, in preparation). In this report, we consolidate our arguments for the "pre-translational" code *via *a closer analysis of:

1. The fundamental veto on "foresight evolution"

2. The recently updated library of direct RNA-aa binding sites --- possibly pointing at the earliest "(stereo chemical) era" in the history of the genetic code [12], and

3. Some features of the genetic code *per se *(Figure 1A), as well as its adaptors (tRNAs) and implementers (aaRSs).

## Results and discussion

### Origin of the genetic code: the major question

Even before the final deciphering of the genetic code, the two general ideas were already aboard with respect to the code's possible origin(s). The two are radically different when it comes to the veto on "foresight evolution". The first ("key-lock") idea assumes some sort of direct stereochemical affinity between amino acids and oligonucleotides that are similar to, or just contain, anticodons (or codons) [13-16]. Apparently, such an affinity does not depend on translation itself, and so life might have used it for pre-translational coding regardless of potential foresight adaptations. This observation leaves open the question of the immediate use of coding without translation, however (see below for candidate explanations).

This is clearly not the case with the other ("adaptor") idea, stipulating existence of intermediate molecular agents capable of recognizing both an amino acid and a corresponding codon in mRNAs simultaneously [17]. The logical difficulty with this idea is that it does not really address the issue of the code origin --- it simply "passes the buck" to the aforementioned adaptors. Now we know that these adaptors are not in the least hypothetical --- they do exist, they are tRNAs (Figure 1C), and it is these molecules in which one usually looks for the clues towards elucidation of the code origin.

However, the anticodon and the site of aa attachment are located on the opposite "poles" of the tRNA molecule (Figure 1C); therefore, its self-aminoacylation seems very unlikely. Not surprisingly, twenty aaRSs, one for each aa, perform this (aminoacylation) function, thus actually implementing the code. Furthermore, since aaRSs are proteins, we are faced here with the proverbial "chicken-or-egg" paradox that necessarily suggests that primordial tRNAs have been aminoacylated by r-aaRSs, the ribozymic iso-functional precursors of protein aminoacyl-tRNA synthetases (p-aaRSs). These putative r-aaRSs have one obvious advantage over p-aaRSs in distinguishing right tRNAs from wrong ones - the easy and precise (through complementary base pairing) recognition of anticodons. This said, the r-aaRSs must have recognized the particular amino acid, and it would seem logical to position the recognition site as close as possible to the tRNA 3' end (in the r-aaRS-tRNA complexes). And so, the central problem remains: a tRNA, in its turn, "passes the buck" to the coding-prone sites of aa-RNA interactions in r-aaRSs. Furthermore, the r-aaRSs, in order to aminoacylate cognate tRNAs, would have required the catalysts of their own, i.e., "meta-r-aaRSs", which situation, in turn, would inherit the same problem and require the catalysts of their own, etc... *ad infinitum *[18-20].

Two important discoveries further exacerbate the problem. First, the p-aaRSs appear to be divided in two distinct classes, their structures having nothing in common (on all, 1D, 2D and 3D, levels). However, they show sterically mirror modes of tRNA recognition: from minor and major groove sides of the acceptor stem [21,22] (Figure 1A). Most likely, this complementarity in tRNA recognition of class I and class II p-aaRSs reflects structural complementarity of their genes --- the complementarity that becomes obvious in the anti-parallel (head-to-tail) alignment and, in turn, might point to their sense-antisense (SAS) origin from complementary strands of one and the same gene-ancestor [23,24].

Second, in parallel with these presumably ancient SAS-based *inter-gene *relationships, both main agents of the code, adaptors (tRNA) and "implementers" (p-aaRS) demonstrate the apparent *intra-gene *modularity. More specifically, for at least ten amino acids, tRNAs truncated to a small piece of the acceptor arm with the CCA3' end have been still able to be charged with a correct aa by cognate p-aaRS [25-27]. Inversely, complete deletion of the anticodon-binding domain in p-aaRSs did not deprive them of the ability to link tRNAs with the anticodon-specific aa (ibid). This amazing anticodon-independent (but still correct) aminoacylation was interpreted as evidence of there being a second (RNA operational) code [26,28] embodied mainly in the first three-four base pairs of the acceptor stem and the unpaired base-determinator.

This operational code of tRNA aminoacylation might have been older than the anticodon-mediated ability to read codons in mRNAs [26], and it is certainly older than the partition of aaRSs in two classes since ten "anticodon-independent" amino acids are evenly represented between them: Val, Ile, Met, Tyr, Cys - class I and Gly, Ala, Ser, Asp, His - class II. In fact, this necessarily implies that the operational code has been initially implemented by the ribozymic precursors of aaRSs. This, in turn, indirectly supports the hypothesis that the classic genetic code (associated in tRNAs with the anticodon) might be a "frozen accident" [29]. At any rate, all of the above does not eradicate the thesis that "at some early stage in the evolution of life the direct association of amino acids with polynucleotides, which was later to evolve into the genetic code, must have begun" [15]. Consistently, in aminoacylation of tRNAs, p-aaRSs first recognize and select a particular amino acid, and only after that the aa-specific tRNAs. Most likely, p-aaRSs inherited this order of recognitions from r-aaRSs, along with many other features [19,30].

The aa-binding sites of r-aaRSs might reflect this primary code. The fundamental question is: Does this putative pre-translational code have anything in common with the actual genetic code used in translation? This question, again, "passes the buck" back to the main logical dilemma: whether the codon-to-aa assignment (Figure 1A) evolved from the aa-binding sites of r-aaRSs, or if it was, indeed, a frozen accident. The *in vitro *selected aa-binding RNAs [12,31] might just provide an answer.

### RNA-amino acid binding sites: Asymmetry in frequency of anticodons and codons suggests pre-translational origin of the genetic code

By now, aa-binding RNA aptamers have been successfully "selexed" for eight amino acids. According to [12], their aa-binding sites are of the three types:

- Sites in which cognate codon and anticodon are both significantly over-represented, this group including arginine, isoleucine and (borderline significant) tyrosine.

- Sites in which only cognate anticodons are found in significant excess, this group including histidine, phenylalanine and tryptophan.

- Sites in which neither anticodons nor codons significantly dominate, this group including glutamine and presumably leucine.

**Figure 2:**
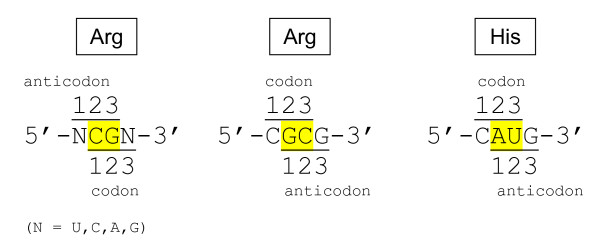
**The palindrome dinucleotide-based overlapping of codons and anticodons expected in aa-binding sites of RNA aptamers**. Shown on the left is the CG-based similarity of arginine codons (CGN) and anticodons (NCG). Self-complementarity of the CG dinucleotide increases probability of finding codon in an Arg-binding site, if anticodon is already there, and *vice versa*. The same is the case for four other amino acids that have palindromic dinucleotide-containing codons: AUN (Ile, Met), UAY (Tyr), GCN (Ala). Shown in the middle is a particular codon of arginine, CGC, which, being CG palindrome-containing at 1-2 positions, is simultaneously GC palindrome-containing at 2-3 positions. Accordingly, if the next nt is G, one gets the anticodon with the same palindrome GC at 1-2 positions. Thus, one and the same tetraplet, CGCG, appears as a codon(1-2)/anticodon(2-3) for palindrome CG, and simultaneously as a codon(2-3)/anticodon(1-2) for palindrome GC. In contrast, histidine's codon CAU (shown on the right) has AU palindrome at 2-3 positions only, hence its anticodon (AUG) appears with the same AU at 1-2 positions.

Initially, it was thought that codons were overrepresented in such RNA aptamers, although inspection of the sequences revealed that when there were codons, anticodons were also there almost invariably [10]. The updated compilation [12] totals 337 RNA aptamers and yet, remarkably, the group of "codons-only" sites remains unpopulated. Looking for possible explanation, we have noticed that in the first (codon + anticodon) group, codons for Arg, Ile, and Tyr have a self-complementary palindromic dinucleotide at 1-2 positions, namely: **CG**N for Arg (N is U,C,A,G), **AU**U, **AU**C, and **AU**A for Ile, and **UA**U and **UA**C for Tyr. Accordingly, the anticodons have exactly the same dinucleotides, but at 2-3 positions, namely: N**CG **for Arg (N are A,G,U,C), A**AU**, G**AU**, and U**AU **for Ile, and A**UA **and G**UA **for Tyr. Therefore it is hardly surprising that, for example, inside the Arg-binding site one base-left shift to its codon **CG**N would necessarily bring us to its anticodon N**CG**. And, the opposite way around: presence of a N**CG **anticodon predetermines finding a **CG**N codon one-base shifted to the right (Figure 2). Thus, in this group of aa-binding sites, codons might simply accompany anticodons, or *vice versa*.

To find out which of the two, anticodon or codon, was actually involved in selection of independent aa-binding RNAs, we first noticed that, formally speaking, the concomitant presence of codons and anticodons is also expected in the reverse situation, when codons contain a palindrome-dinucleotide at their 2-3 positions, whereas anticodons exhibit them at 1-2 positions, i.e. as if the codons and anticodons switched their places. Figure 2 shows an example: His codon C**AU **and anticodon **AU**G.

Before we move further, we will denote these two cases of palindromic dinucleotide-containing coding motifs as codon(1-2)/anticodon(2-3) and codon(2-3)/anticodon(1-2), or, for simplicity, (1-2)/(2-3) and (2-3)/(1-2), respectively. Combination of both of these cases is also possible (see, for example, CGCG tetraplet in Figure 2).

In the complete code (Figure 1A), a number of codons with any such palindrome at 1-2 positions is equal to that with the same palindrome at 2-3 positions. One would then think that these two perfectly symmetrical cases, (1-2)/(2-3) and (2-3)/(1-2), should have equal chance to be found in aa-binding sites of selexed RNA aptamers. Indeed, there are no reasons whatsoever for the *a priori *belief that the procedure of RNA aptamers selection *per se *--- the selection focused on stereo-specific binding of amino acids to particular RNA sequences --- has anything to do with translation (hence, by the way, the wobbling interface between codon and anticodon at their 3^rd ^and 1^st ^nts, charging tRNAs with cognate amino acids by p-aaRSs, etc.)

However, Table 1 in [12] unambiguously shows that the codon(1-2)/anticodon(2-3) cases, but not their codon(2-3)/anticodon(1-2) "counterparts", are over-represented at significant level in Arg-, Ile- and (with borderline significance) Tyr-binding sites. A closer inspection of aa-binding RNA aptamer sequences for these amino acids uncovered a number of fascinating details. As the control data, we used His- and Leu-binding RNAs, because their codons contain (just at (2-3) positions), the AU and UA palindromes: C**AU **(His) and U**UA**, C**UA **(Leu).

#### Arginine

In the set of 34 Arg-binding sites, NCG anticodons predominate, often together with overlapping CGN codons; the most frequent motifs being 5'-UCGA-3', 5'-UCGC-3' and 5'-GCGG-3'. Importantly, *these three are all of the codon(1-2)/anticodon(2-3) type.*

The motif 5'-CGCG-3' is of a special interest (Figure 2), because one can see it in two ways: CGC is a codon(1-2) for CG palindrome and, simultaneously, it is also a codon(2-3) for GC palindrome. The overlapping GCG anticodon, in its turn, can be considered as the (2-3) or (1-2) one for CG and GC, respectively. This ambiguity is fraught with confusion for coding. Very telling, therefore, is the fact that Arg-binding sites *do not contain the 5'-CGCG-3' motif at all, in contrast to the above three motifs of the codon(1-2)/anticodon(2-3) type. *This fact becomes even more telling if we take in account presence of 5'-CGCG-3' beyond aa-binding sites ([12]: see, in the Arg list, cases 17, F2e, F2f, and F2U).

Overall, anticodon UCG significantly outnumbers CGN codons, suggesting that codons might simply follow anticodons (like hitch hikers) rather than *vice versa*. The only exception is codon AGG (see [12]). However, among the 34 sites, there is not a single one in which AGG appears as itself, without concomitant NCG and/or CGN cognate triplets. Moreover, in the latest compilation set of 127 newly isolated smallest Arg-binding sites [31], the only significantly over-represented triplet is the AGG's complement, arginine's anticodon CCU. Thus, *when for the same amino acid, arginine, we find in its RNA-binding sites a cognate*, ***but not-CG-containing****, triplet, it appears to be the anticodon, not codon.*

#### Isoleucine

The available set of Ile-binding RNA aptamers is the most populous one: 185 sequences. Ile's three codons have a palindrome, AU, at 1-2 positions, and one of these three, AUA, also has another palindrome, UA, at 2-3 positions.

**Figure 3:**
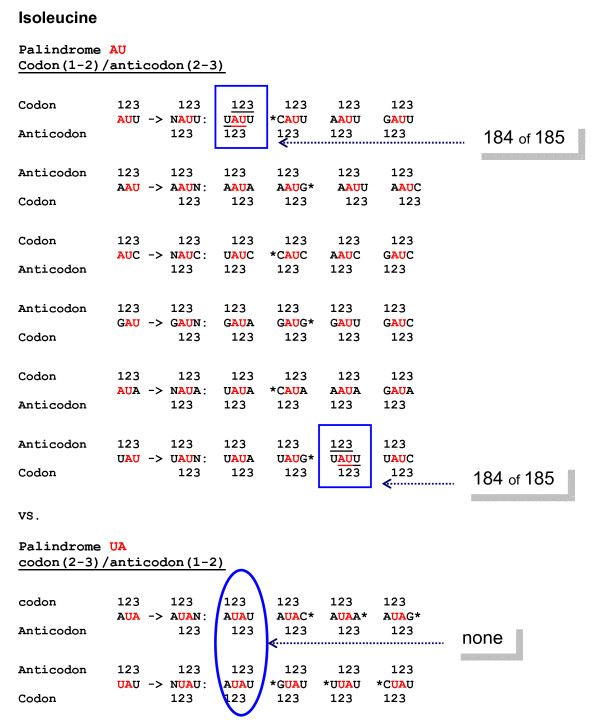
**All conceivable tetraplets which are supposed to occur in Ile-binding sites if one of its coding triplets, either AUU, AUC, AUA (Ile codons) or AAU, GAU, UAU (Ile anticodons) is already there**. These tetraplets consist of codon and anticodon overlapping at the palindrome-dinucleotide. Marked by asterisk are tetraplets in which codon or anticodon represents a different (not Ile) amino acid. **A**. The tetraplets expected for palindrome AU at codons' 1-2 positions and anticodons 2-3 positions, respectively. Shown boxed is the tetraplet 5'-U**AU**U-3' which one can actually see in all Ile-binding sites of independently selexed RNA aptamers (184 of 185 cases). Remarkably, this highly conserved motif constitutes the Ile-specific internal loop that seems to be directly involved in function [93]. **B**. The tetraplets expected for palindrome UA at codon 2-3 positions and anticodon 1-2 positions. Shown in oval is the ile-specific tetraplet 5'-A**UA**U-3' which one would expect to observe if the UA palindrome in overlapping anticodon UAU and codon AUA was favored by selection of RNA aptamers specifically binding Ile. Remarkably, in contrast to ubiquitous 5'-U**AU**U-3' in the group A (codon(1-2)/anticodon(2-3) tetraplets), none of Ile-binding sites contains the motif 5'-A**UA**U-3' in seemingly symmetric group B (codon(2-3)/anticodon(1-2) tetraplets).

Figure 3 shows the tetraplets which are supposed to occur in Ile-binding sites if one of its coding triplets, either AUU, AUC, AUA (codons) or AAU, GAU, UAU (anticodons) is already there. Out of this extensive repertoire, only one (5'-U**AU**U-3') tetraplet does occur (and in obvious excess) in numerous independently selected Ile-binding sites. Note that this motif belongs to the (1-2)/(2-3) group. The symmetry of (2-3)/(1-3) motifs suggests competitive presence of the 5'-A**UA**U-3' tetraplet, in which A**UA **can be considered as the Ile's codon(2-3), and the overlapping **UA**U as the Ile's anticodon(1-2), respectively. However, the whole set of 185 Ile-binding RNA sites (2508 nts in total) does not contain even a single 5'-AUAU-3'. In contrast, beyond the sites (9915 nts), we can see 28 such tetraplets, one per 354 nts, on average. Proportionally, by chance alone, one would expect to find seven tetraplets 5'-AUAU-3' in Ile-binding sites. Its complete absence may hint at the selection acting against it. A strong independent evidence for such selection comes from Tyr-binding sites.

#### Tyrosine

One of tyrosine's codons, UAU, is a (precise) complementary partner of isoleucine's AUA. Only three Tyr-binding RNA aptamers are available [12]. However, these three display exactly the same pattern, as do much more representative Arg- and Ile-binding ones: the significant excess of 5'-A**UA**C-3' tetraplet, which consists of tyrosine anticodon A**UA **overlapping with codon **UA**C. This is, again, a motif of the codon(1-2)/anticodon(2-3) type. The seemingly equivalent 5'-U**AU**A-3' of the codon(2-3)/anticodon(1-2) type is not to be found in Tyr-binding aptamers.

It should be emphasized that the 5'-A**UA**C-3', and its closest homolog 5'-A**UA**U-3', are the very motifs that belong to the codon(2-3)/anticodon(1-2) group for isoleucine. However, neither is located in the Ile-binding sites (see Figure 3). Instead (and this is highly unlikely to be just a coincidence), we find the 5'-A**UA**C-3' precisely where the codon(1-2)/anticodon(2-3) "rule" predicts: in binding sites of the Ile's complementarily encoded partner --- Tyr. It would be hard to find a more convincing argument for the preference of (1-2)/(2-3) tetraplets over seemingly symmetric (2-3)/(1-2) in aa-binding sites of selexed RNAs.

#### Histidine

This amino acid shows an excess of its GUG (highly significant) and AUG (borderline significant) anticodons in aa-binding sites of selexed RNA aptamers [12]. What makes histidine important for our analysis is the fact that one of its codons, C**AU**, happens to be of the (2-3) type, the anticodon **AU**G being, complementarily, of the (1-2) type. Therefore, were the (1-2)/(2-3) and (2-3)/(1-2) motifs had had equal chances to be selected in aa-specific RNA aptamers, the His's (2-3)/(1-2) motif 5'-C**AU**G-3' would be found in significant excess within the His-specific binding sites. However, among such sites (54 independently selexed RNAs) we do not see even a single 5'-C**AU**G-3' --- the absence being all the more telling if one takes into account the fact that this tetraplet is quite common *outside *of the His-binding sites.

#### Leucine

According to Yarus et al. [12], concentration of cognate triplets in the aa-binding sites of the independently recovered (15 times) Leu-specific RNA is not significant. Yet, it is the Leu's C**UA **codon(2-3), which is represented by its **UA**G anticodon(1-2) (with borderline significance); and again, exactly as with His's C**AU **codon (see above), one observes the corresponding (2-3)/(1-2) tetraplet 5'-C**UA**G-3' *outside, but not within*, the Leu-binding site (ibid).

In summary, we point out that:

1) Anticodons, not codons, are more often significantly over-represented in aa-binding sites of the RNA aptamers.

2) For amino acids encoded by dinucleotide-palindrome-containing triplets, their binding sites in RNA aptamers "prefer" the codon(1-2)/anticodon(2-3) motifs over the codon(2-3)/anticodon(1-2) ones in spite of their seemingly perfect symmetry.

These striking preferences mean that the 3^rd ^nt is more important than 1^st ^nt in anticodons (complementarily, the reverse being the case with codons), *precisely as in the real genetic code *(Figure 1A). However, since selection of aa-specific RNA aptamers apparently had nothing to do with translation, it would be more correct to say that in the interface between interacting aas and cognate triplets, the 2-3 nucleotides contribute more to the specificity of interaction, *thus determining their future usage as an ****anti****codon*. Furthermore, selection of aa-specific RNA aptamers had nothing to do with the translation machinery (in contrast to primary coding). If so, the revealed strong bias towards anticodon's 2-3 (and, complementarily, codon's 1-2) palindromes has been established very early, before translation, i.e., before the codon-anticodon interaction in tRNA-mRNA complexes. Consequently, not only the translation machinery itself, but its coding toolkit, the code-adapting molecules (tRNA) and aaRSs (initially, advanced ribozymes, then minimalist primitive enzymes) have been evolving to ''fit'' the probably already basically outlined genetic code (as opposed to the code co-evolving with tRNAs and aaRSs to "fit" translation by minimizing translation errors).

Does this translation-independent preference of (1-2)/(2-3) over (2-3)/(1-2) triplets suggest some fundamental left-right, chirality-like, asymmetry, and if it does, could this asymmetry determine the salient features of coding and translation? Our (guarded) answer is Yes. However, the code's primordial adaptors (tRNAs) and implementers (r-aaRSs) could have simply used the preexisting aa-triplets' affinities in the way that minimized errors of aminoacylation. That is, the original aa-triplet preferences within the aa-binding sites of RNA catalysts determined the primal pre-translational genetic code with more important 2^nd ^and 3^rd ^nts, whereas 1^st ^nt was much less specific. Later, when the code was expanding in co-evolution with the translation apparatus, the importance of 2-3 nts of coding triplets "passed" on 1-2 nts of their complements thus distinguishing anticodons from codons. The fact that codon's 3^rd ^nt is more degenerated than the anticodon's 1^st ^nt actually serves as an indirect evidence of in support of this order of events.

### Imprints of the pre-translational code in tRNAs: One ancestor for two codes

If the code did have a pre-translational origin, one would look for its imprints in the code adaptors, tRNAs. The very structure of tRNAs is quite interesting in this regard. Indeed, anticodons are directly involved in "reading" mRNAs *via *recognition of complementary codons, but the anticodon-to-aa association (the code *per se*) is indirect, provided solely by the physical linkage of the aminoacylation and codon-reading sites within a tRNA molecule. This is a crucial point on which, among other things, the idea of frozen accident [29] hinges upon, and the anticodon-free charging of many tRNAs with cognate aas, i.e. the operational code of tRNA aminoacylation, might still reflect this ancient fortuity [26]. At the same time, new data on riboswitches in general [32], recent discovery that in the ribosome the anticodons are selectively enriched just in proximity to their amino acids [33], an excess of the aa-respective anticodons just in the aa-binding sites of in vitro "selexed" RNA aptamers [12], and our own study of these sites (see above) revealing the striking bias of triplets with dinucleotide palindromes at 2-3 positions towards the real anticodon-to aa assignment - all these facts suggest that the hypothetical ancient pre-translational code and the real pro-translational code are in fact one and the same code.

Furthermore, the pre-translational stereo-chemical hypothesis for the code origin [12,16] and the hypothesis of the ancient operational code preceding the canonical one [26] do not contradict but might, in fact, strengthen each other if the acceptor arm is older than the anticodon arm, but both have a common ancestor.

It seems reasonable to suppose that hypothetical r-aaRSs recognized proto-tRNAs by complementary base pairing. This means that they looked like tRNA complements additionally endowed with the aa-specific aminoacylating activity [34]. Accordingly, not just tRNAs but rather *pairs *of tRNAs with complementary anticodons could still retain the imprints of the code's earliest (pre-translation) history. Our three previous findings support this statement:

1) Pairs of tRNAs with complementary anticodons turn out to be complementary at the 2^nd ^base of the acceptor stem as well [20,35]. To the best of our knowledge, this concerted dual complementarity remains the only evidence (however indirect) for the anticodon and first three bases of the acceptor stem possibly having a common ancestor.

2) Two sterically mirror modes of tRNA aminoacylation might have prevented ancestral tRNAs with complementary anticodons from (otherwise highly probable) confusion [19,36]. The "rules" that determine which pairs of complementary anticodon loops should be recognized from the same, and which ones from the opposite, sides in order to avoid their confusion (Figure 1B) seem to have been implemented very early, most likely by ribozymic precursors of the two enzymes (ibid).

3) However, structurally simplest, presumably earliest and complementarily encoded amino acids such as Gly and Ala seem to never had any risk of their tRNAs confusion depending on the anticodon loop, no matter whether the putative r-aaRSs recognized the two complementary halves of tRNAs (Figure 1C) from the same or opposite sites [20]. The high risk of confusion did take place for proto-tRNAs of such amino acids, but for recognition of their acceptor arms, not anticodons, the acceptor's second base pair playing the crucial role in this (ibid).

**Figure 4:**
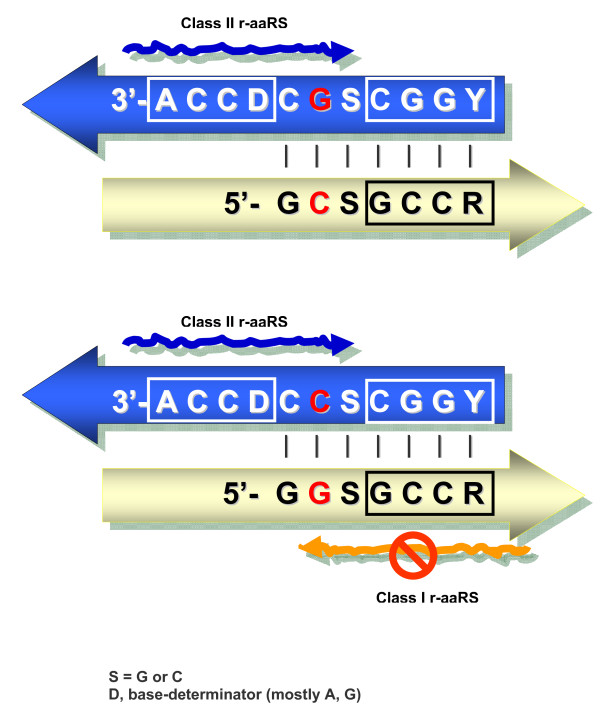
**Ancestral acceptor arms of tRNAs with complementary second base pairs (shown in red) and putative r-aaRSs of two types shown as wavy arrows**. The 3' × 3' (blue × blue) variant of recognition of acceptors is much less confusion-prone in comparison with the "prohibited" 5' × 3' (blue × yellow) one (adopted from [20]).

Figure 4 reveals a possible reason. It shows a common ancestor of acceptor arms, reconstructed from the phylogenetic trees of Bacteria, Archaea and Eukarya. The acceptor's 5' strand consists of a triplet that might represent, according to the dual complementarity, a proto-anticodon and a quadruplet GCCR that is homologous to the universal DCCA 3' tail. Consequently, the 3' strand of the acceptor emerges as a short palindrome (11 nts) with a codon-like triplet in the middle. It is easy to see that in order to avoid confusion between two acceptor stems with complementary second bases (as it is in the case of Gly and Ala), the aminoacylating ribozymes must use the same groove side; otherwise, they would actually encounter identical targets of recognition. Of two such variants, the 3' × 3' (blue × blue) looks more advantageous than the 5' × 5' (yellow × yellow) due to the unpaired DCCA-3' tail. And it is this variant which has been really chosen despite the fact that within the anticodon loop three other variants (5' × 5', 5' × 3' and 3' × 5') were equally error-proof (see [20] for detail).

Worthy of note, alanine is one of five amino acid encoded by the palindromic dinucleotide-containing triplets at 1-2 positions, GCN. It would be quite interesting, therefore, to test ala-binding sites of RNA aptamers on the (1-2)/(2-3) over (2-3)/(1-2) preference. However, alanine, as well as its complementary partner glycine and all other blue × blue pairs of "Miller's" amino acids (Pro, Thr, Ser), are absent in the library of aa-binding RNA aptamers [12]. Insensitivity of their tRNA recognition modes to anticodon loops and, on the contrary, a strong association of the confusion risk with the acceptor arm (Figure 4) may turn out to be very meaningful in this connection.

### Origin of tRNA: From a proto-anticodon and a 5'-DCCA-3' to a cloverleaf

**Figure 5:**
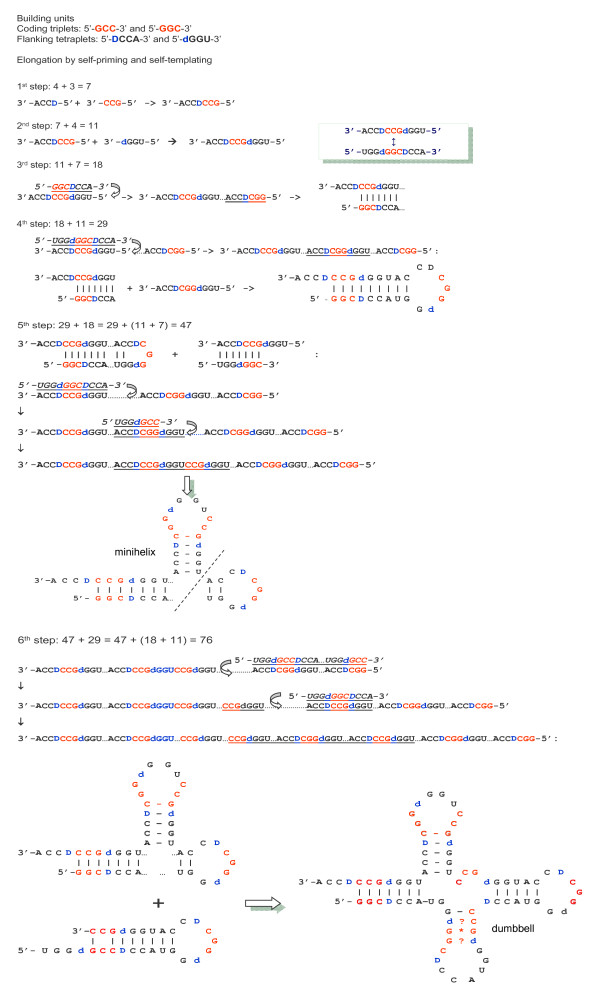
**The model of consequent "quasi-Fibonacci" growth of tRNAs from proto-anticodon triplet and 5' DCCA3' quadruplet to the final cloverleaf-shaped molecule (see also **[20]**and **[35]).

Pre-translational origin of aa-specific anticodons is consistent with our model of tRNA growth (Figure 5). We have noticed that the two coding units, a triplet (presumable proto-anticodon) and DCCA (presumable tag of replication with a coding base-determinator D) would eventually generate the 76 nt-long cloverleaf if we will use them as building bricks in the Fibonacci-like iterative process. Literally: 3 + 4 = 7, 4 + 7 = 11, 7 + 11 = 18, 11 + 18 = 29, 18 + 29 = 47, and finally 29 + 47 = 76!

This algorithm produces the cloverleaf with periodically repeated ACCR and YGGU motifs and many of the conservative and largely conservative bases, such as 5'-URG-3' at 8-10 positions, the anticodon flanking 3'-U and R-5' bases, the 3'-C of the V-loop which is homologous to the nearly invariant 3'-C at the 72^nd ^position (see also: [20,35]).

Importantly, in order to arrive at a final "perfect" cloverleaf, one should, in each step "i" of the elongation process (i = 1, 2,...,6), add to the (i-1)th member not just the preceding (i-2)th one, but its complementary image. This actually implies that during the code development the repertoire of aa-specific triplets has been increasing not one by one but rather by complementary pairs, in accord with numerous independent arguments [18-20,23,24,34-43]. This also implies a self-priming and self-templating, mechanism of elongation (Figure 5). As a consequence, the 76-nt long cloverleaf would have internal sequence periodicity --- the hallmark of tRNAs reported long ago ([44], see also [35]).

The 11 nt-long precursor is a palindrome, which may serve a template for its perfectly symmetric partner with a complementary triplet in the center (Figure 5; step 2). Two other structures, the 29 nt-long hairpin (step 4), and the eventual cloverleaf (step 6), also show such complementary symmetry (although less perfect) with the anticodon mapped right in the middle. (By the way, the final cloverleaf and its 47 nt-long precursor can be both represented as a long hairpin, with a few small bulges, as well (not shown).) Indirectly, this symmetry also supports the hypothesis that new amino acids entered translation by complementarily encoded pairs ([37], see also [20,35,38,39]).

**Figure 6:**
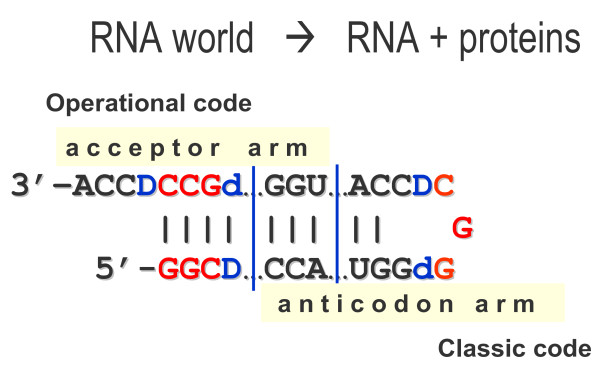
**The 29 base-long precursor of tRNA with overlapping acceptor and anticodon domains**. At this stage, tRNAs become a true adaptor of the code since they have a single-stranded anticodon loop for reading codons in mRNAs.

**Figure 7:**
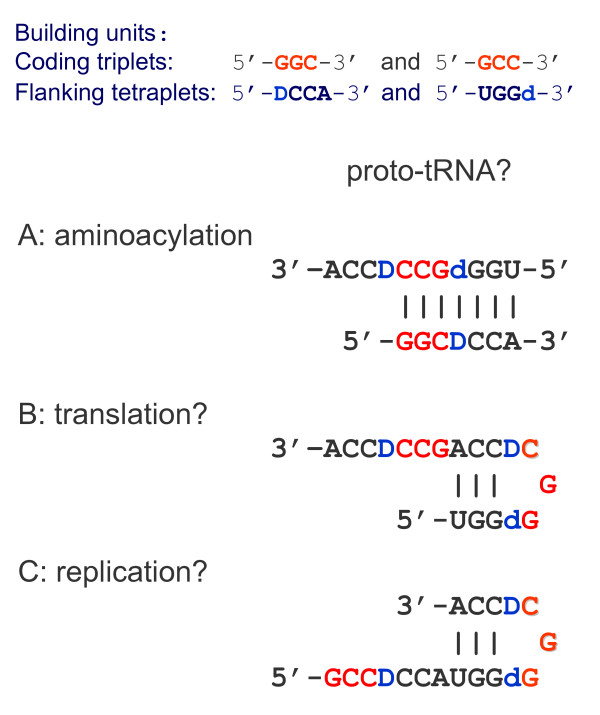
**Possible tRNA precursors: variants of (11 + 7 = 18) elongation**. Variant A represents the canonical acceptor arm. Variant B could also be used in primordial translation. Variant C might represent even the earlier stage of an RNA replisome [45] with pre-tRNAs functioning as donors of trinucleotides for growing complementary RNA strand during replication.

The following aspects of the model are worthy of special comments:

First, tRNAs could participate in translation before they have "reached" the complete cloverleaf. The 29 nt-long hairpin with overlapping acceptor and anticodon domains is an obvious candidate to be such a precursor for code adaptors. tRNA is perhaps the most important molecule of life, bridging RNA and (RNA + Proteins) worlds. As Figure 6 symbolically shows, the two worlds are literally as close as it possibly gets in this hairpin: the late RNA world with the operational code actually joins the early (RNA + Proteins) world with the classic code. In principle, the 18-nt long acceptor arm also assumes two alternative versions of combining the 11- and 7-nt long precursors usable in translation (Figure 7B) or even earlier - in replication (Figure 7C) with the ribosome functioning as an RNA replisome and tRNAs loaded with trinucleotides (not amino acids) and donating them to growing complementary RNA molecules ([45], see [4,5,46,47] for more comprehensive treatment).

Second, if the primordial operational code of aminoacylation involved many amino acids, the question arises how their short proto-tRNAs could evolve in unison, towards the same final shape [35]. The origin of tRNAs might have been non-monophyletic, indeed [48] --- thus making the above question critical for any model of the tRNA origin from a smaller precursor. The process of Fibonacci-like elongation is quite specific in this regard: starting from the 5'DCCA3' quadruplet and different (including complementary) coding triplets, it brings them all to the universal 76 nt-long cloverleaf. Of course, any elongation that took place with a single proto-tRNA could then be spread across the whole repertoire of already existing code adaptors by a variety of mechanisms (see [49] for review), but the Fibonacci-like elongation obviously makes such "coincidental coevolution" [50] much easier.

**Figure 8:**
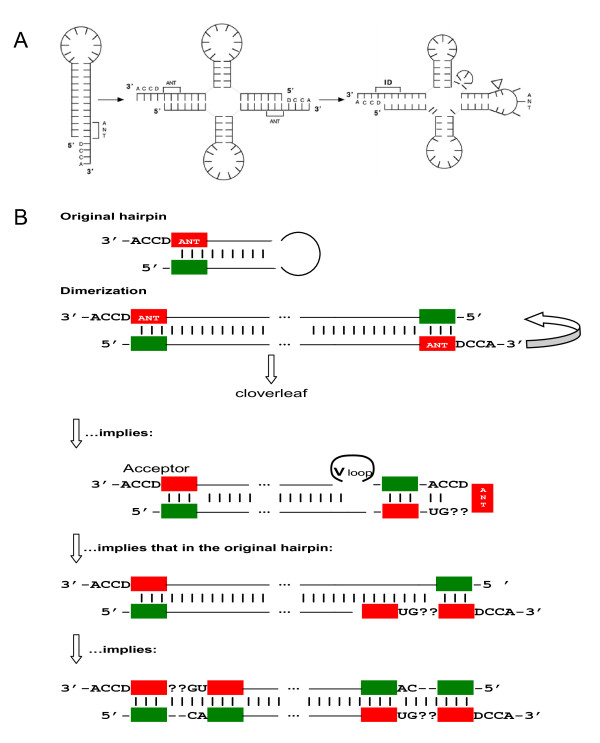
**The Di Giulio's model of tRNA origin by dimerization of hairpins**. **A**: The model [51,52]. The anticodon is supposed to be located on the 3' strand of the hairpin just before the terminal 5'DCCA3' motif. **B**: The scheme showing that the Di Giulio model necessarily suggests internal duplications in original hairpins. The anticodons are shown red, their complements - green.

Third, our model (Figure 5) may seem incompatible with the Di Giulio model (Figure 8A), in which tRNAs originated from a hairpin with a proto-anticodon in the 3' strand preceding the unpaired DCCA tail. Two such hairpins make a cloverleaf if one is shifted along the other with formation of the unpaired anticodon loop (and, as a consequence, V loop) as shown in Figure 8A[51,52]. In this simple and generally elegant model, the anticodon precursor is positioned in the same place as in our model --- adjacently to the 5'DCCA3'. However, in the Di Giulio model, the anticodon and its putative acceptor's precursor are supposed to be identical whereas in our model they are complementary to each other. Note that in consensus and ancestral tRNAs, at the purine/pyrimidine (R/Y) resolution, second bases of anticodon and its presumable precursor in the acceptor are much more often complementary rather than identical [20,35]. Moreover, for the amino acids which (in the majority of origin scenarios) are supposed to be the very first ones recruited by emerging code and translation, i.e. Gly, Ala, Asp, Val, Glu, their tRNAs all have complementary second bases in the anticodon and 3' strand of the acceptor stem. The same is the case for two tetrads, [Ala (GGC), Gly (GCC), Val (GAC), Asp (GUC)] and [Ala (CGC), Arg (GCG), Val(CAC), His (GUG)], which might have formed the complementary core of the genetic code (see [18,19,40] for details). And, in convincing contrast, the tRNAs which are consistent with the "dimerization" model (Figure 8A), all represent relatively "late" amino acids: Leu, Gln, Trp, Phe and Cys.

Yet, a closer analysis of the "dimerization" model (Figure 8A) reveals its similarity with the model proposed here (Figure 5). Indeed, Figure 8B clearly shows how shifting of the 3' half of one hairpin forms the anticodon loop and, consequently, V-loop, but necessarily implies preexistence of the anticodon duplicate (plus UG-3') intended to complement the codon triplet (plus 5'-CA) on the 5' half of the opposite hairpin. Symmetrically, this anticodon duplicate in turn suggests preexistence of the codon duplicate in the original hairpin, etc., - in all, we get eight coding triplets, four anticodons and four codons, each with adjacent UG or CA dinucleotide, respectively - precisely as in our model (Figure 5). It thus turns out that "simple" dimerization of two identical hairpins with anticodon/codon-like pair at the basis generates the tRNA cloverleaf with the unpaired anticodon in the center if and only if the hairpin itself originated by complementary duplications of shorter precursors. Actually, it is the self priming and self templating model of tRNA growth [44] that is at work here, and our model (Figure 5) is its Fibonacci process-based version (see also the close variant in [20]). Among other similar versions (not shown), we would like to mention here the simple series of six duplications of the original palindrome (of alternate complementarity) producing the final 73 nt-long cloverleaf (11 × 7 less the 5' terminal dGGU tetraplet) and the model of three duplications of the acceptor arm with the additional base "d" on the 5' strand, which is complementary to the base-determinator "D" on the 3' strand. In this (19 × 4 = 76) model, the intermediate (19 × 2 = 38) state is in fact equivalent to the primordial tRNA hairpin in the Di Giulio's model (Figure 8A). The (19 × 4 = 76) model suggests also that ancestral tRNAs might have had in the acceptor arm a complementary intron preceding the "d" and, indeed, the majority of tRNAs of histidine do contain such complementary base-determinator. (Interestingly, His is the amino acid of highest catalytic propensity [11].)

Fourth, in addition to the typical 37/38-positioned intron in tRNA genes [53-56], the recent *in silico *genome-wide search for tRNAs in some archaebacteria and eukaryotes unexpectedly discovered the great diversity of disrupted tRNA genes (reviewed in [57]). The collection of encoded-in-pieces tRNAs with an intron at 37/38 site is enriched by the genes with a single intron at non-canonical positions [58,59], the genes with multiple introns [60], the split genes that separately encode the 5' and 3' halves of tRNAs [61-63], the tri-split tRNA gene precedents [64,65], and permuted tRNA genes, in which the sequence coding for 3' half precedes that for the 5' half [66,67].

This impressive diversity is precisely what any model of gradual tRNA elongation predicts (e.g. Figure 5) because the anticodon-flanking bases come actually from the primordial palindrome (Figure 4) as homologs of the base-determinator "D" and its complement "d". It does not mean, of course, that the entirety of the tRNA splitting diversity that we observe today directly descends from the corresponding intermediate stages of tRNA growth; certain present-day disrupted tRNA variants might well be the "analogs" rather than the homologs of the putative tRNA precursors - analogs that readily emerge simply due to the revealed multiple modularity of a tRNA molecule. The "homology vs. analogy" uncertainty here is similar to that encountered with the origin of deviant codes (see [68] for review); while the phylogeny tells us that they must be late deviations, we do know that there must have been such codon-to-aa variations during the origin of the universal genetic code as well. And yet, it has not escaped our attention that, most frequently, D is A, d is U, and it is again the invariant A (sometimes G) and U that flank the anticodon from the 3' and 5' sides, respectively.

There is a certain rationale behind the fact that the major site of introns, splits on minigenes and processing in permuted tRNA genes is located between the 37^th ^and 38^th ^nts. It is this position that is consistent with the sub-code for two modes of tRNA aminoacylation (from major and minor groove sides) - the sub-code that minimizes the risk of confusion for pairs of tRNAs with complementary anticodons [19,36], especially if we put the putative aminoacylating ribozymes in introns, in proximity to the 37^th ^base [9,10,69]. Furthermore, the sub-code might have already worked in shorter precursors such as the 29-nt long proto-tRNAs with the presumable site of intron also mapped right in the middle (between 15^th ^and 16^th ^nts), or even 11-mer precursors, right after the base-determinator D.

According to Di Giulio [48,52,70], the most frequent intron position right in the middle (37/38) of a tRNA molecule reflects its origin by hairpin dimerization (Figure 8A). In contrast, as far as other flexible sites of tRNA disruption are concerned, Di Giulio [71] explains away all such cases after Randau & Söll [61-63,72] - due to gained protection against integrative mobile elements. However, the "main" and "peripheral" introns are processed uniformly, and they are undistinguishable in everything but location. Therefore, like Randau and Söll [73], we do not find these two explanations readily compatible. At the same time, the purely "protectionist" hypothesis of Randau and Söll does not appear to be entirely convincing, either (see [71]).

There is, however, no need for any *ad hoc *explanations of the sort if we accept the models of multi-step self-primed and self-templated elongation of tRNAs. For example, in our model (Figure 5), the 37^th ^base has a series of precursors starting from the base-determinator D in the original heptamer and 11-mer palindrome. Remarkably, the additional sites of tRNA disruptions are often mapped right next to, or one-two bases shifted from, the "D" and its complementary "d" bases, following or preceding them, respectively. Thus, our model (Figure 5) uniformly completes the evolutionary interpretation of all tRNAs-in-pieces: not only the 37/38-positioned hot spot of tRNA splits but also the majority of "warm" and "cold" spots might reflect the deep evolutionary past of tRNAs marking crucial steps in their development from a coding triplet and DCCA up to a final cloverleaf. In our model, the 37/38 position of intact tRNAs is identical to similar positions of shorter precursors (like the 15/16 position in the 29-base long proto-tRNA) as if it "inherited" all previous stages of tRNAs elongation thus, accordingly, becoming the major site of possible disruptions.

However, in our opinion, neither the interpretation limited to the 37/38 site, nor its extension to all other disruption sites in our model are incompatible with the Randau's and Söll's "anti-parasitic" explanation. After all, consistently with the "homology vs. analogy" proviso, we do not see why the preferable targets of intron (or mobile element) insertions could not coincide with the junction sites in the step-by-step growing tRNAs - rather, this is where one would expect to find them, right at the "critical points of growth".

## Conclusions

Recently, we have pointed out that probably long before the origin of translation the genetic code had already fixed such a fundamental (and, one would think, translation-ineradicable) feature as the tripletness of coding words [20,24]. The present analysis of aa-binding sites in selexed RNA aptamers allows us to make an even stronger conclusion: the degeneracy of 1^st ^position in anticodons (3^rd ^position in "future" codons) also appears to have originated before translation.

This conclusion questions many dogmas of the code and translation origins, including the almost commonly shared view that the code shaping was driven by minimization of translation errors and, in general, the code structure reflects its coevolution with tRNAs and aaRSs. Not completely denying such translation-motivated coevolution, we would like to accentuate the reverse causality. The very logic of tRNA evolution from a short ancestor to the eventual functionally sophisticated cloverleaf-like molecules might have been dictated by the "RNA-aa binding: a stereochemical era for the genetic code" [12]. Consistent with this are our model in which the coding triplet and DCCA 3' end for aminoacylation are present from the very beginning (Figure 5) and previous findings: the dual complementarity [18,20,35] as an indirect evidence of ancestral anticodon duplication [51,74,75], and the subcode for two modes of tRNA recognition by hypothetical aminoacylating ribozymes (Figure 1B) [19,36]. Apparently, the sub-code for two modes of RNA aminoacylation could be implemented for primordial 11 nts-long palindromes, i.e. extremely early - in response to increasing catalytic and other challenges of the RNA world.

The bias to codon(1-2)/anticodon(2-3) tetraplets in aa-binding sites of RNA aptamers may point to a fundamental interlink between the primordial RNA operational code (hence the genetic code itself) and a chiral selection of its components. Indeed, in model ribooligonucleotides-assisted aminoacylation of RNA minihelices, selection of L-amino acids was determined by a pre-selected D-ribose [76-78]. With a fascinating mirror symmetry, the artificial L-ribose-based RNA system exhibits the clear opposite preference of D-amino acids (ibid). One would then enquire: What if the chiral-mirror RNA world with L-ribose and D-amino acids is just the codon(2-3)/anticodon(1-2) biased? The mirror selexed RNA aptamers could provide an answer.

It should be noted that, in principle, the hypothesis of pre-translational origin of the genetic code is consistent with the hypothesis of its shaping in coevolution with amino acids biosynthesis proposed in [79,80] and extended in [81]. Indeed, if the advanced RNA life did have widely used amino acids (for example as cofactors of ribozymes [9-11]), then it seems reasonable to assume that to some extent the earliest shaping of the genetic code in general, and the order of entering of amino acids into coding in particular, could recapitulate their biosynthetic pathways.

The stereochemical affinity between aa and the anticodon-containing aa-binding sites in selexed RNAs does not preclude, of course, further optimization of codon-to-aa assignment. Very telling in this regard is the sparseness of triplet usage in aa-binding sites [12]: usually for each aa there is only one significantly preferable anticodon (e.g. see Figure 3). Consistently, for most of amino acids the number of anticodons is less than that of codons. This fact points, yet again, to primacy of anticodons, and not codons, in the genetic code origin. Obviously, the repertoire of codons was filled out later, perhaps together with minimization of translation errors in synthesis of very first proteins, such as heat shock proteins, "RNA chaperones" [4,5] and proto-aaRS enzymes ([18-20,22,24,82-86], however, see [7]). And yet, the current analysis shows that the core of the genetic code might have emerged much earlier, certainly before translation.

We propose here a new model of stepwise, Fibonacci process-like elongation of a tRNA molecule from just two (triplet and quadruplet) motifs to a 76 nt-long cloverleaf (Figure 5). Importantly, the earliest part of the molecule, the acceptor arm, already contains the primordial anticodon/codon pair. This means that the two concepts - the pre-translational code and the more ancient operational code of tRNA aminoacylation preceding the classic code for reading mRNAs - may tell actually one and the same story, rather than two different ones. Accordingly, we repeatedly emphasize that the hypothesis of primal stereo-chemical affinity between aas and anticodons (or codons) [12,16], the hypothesis of coding coenzyme handles for amino acids [9,10], the hypothesis of tRNA-like genomic 3' tags suggesting that tRNAs originated in replication [87-89], the hypothesis of the proto-ribosome having served originally a role in RNA replication with the help of primordial tRNA molecules delivering trinucleotides [4,5,45-47] - all four evidently pre-translational - and the hypothesis of the ancient RNA operational code, most likely ribozymes-mediated and pre-translational as well [26], do not contradict but, on the contrary, strongly support each other.

To conclude, our retrospective analysis of aa-binding sites in RNA aptamers and the "proto-anticodon → cloverleaf" model of tRNA growth add to the fundamental premise: *Translation without code does not make sense, but code without (and before) translation does.*

## Methods

The common ancestor of acceptor arms (Figure 4) was reconstructed from the phylogenetic trees of Bacteria, Archaea and Eukarya as detailed in [20]. A combination of manual sequence alignment, Neighbor-Joining phylogenetic tree reconstruction method with Tamura-Nei distances and manual Parsimony-based ancestral state reconstruction was used.

The selected amino acid binding sites, comprising 337 independently derived sequences (18,551 nucleotides in total) directed at eight amino acids, are described in [12]. The curated sequence libraries are available directly from Yarus et al [12].

The statistical significance of over- or under-representation of anticodon/codon triplets was ascertained using chi-square or similar (G-test, exact binomial) two-sided statistical tests.

## Abbreviations

aa: amino acid; nt: nucleotide; tRNA: transfer RNA; p-aaRS: protein aminoacyl-tRNA synthetases; r-aaRS: putative ribozymic precursors of protein aminoacyl-tRNA synthetases; R purine (G or A), Y pyrimidine (C or U); D: base-determinator; d: base complementary to D; SAS: Sense/Anti-Sense in frame coding of two proteins from complementary strands of the same gene.

## Competing interests

The authors declare that they have no competing interests.

## Authors' contributions

ASR contributed to the conception of the study, carried out the analyses, and drafted the manuscript. ES contributed to the conception of the study and helped to draft the manuscript. SNR conceived the study, contributed to the conception of the study, developed the model, and helped to draft the manuscript. All authors read and approved the final manuscript.

## Reviewers' comments

### Reviewer's report 1

Eugene V. Koonin, National Center for Biotechnology Information, National Library of Medicine, NIH, Bethesda, USA

#### Reviewer Comments

This manuscript addresses arguably the most fundamental and most difficult problem in the study of the origin of cells: the steps leading to the emergence of the translation system. Any informed discussion of this fundamental enigma is of interest, and this is particularly true of this exceptionally well written and thoroughly referenced manuscript. I find it equally evident that the decisive breakthrough remains elusive, and even the direction in which one should proceed to find it is less than obvious. So does this analysis take us a step closer to a (the) solution?

In their discussion, Rodin et al. proceed from the unassailable conclusion that p-aaRS must have been preceded by r-aaRS at the early stage of evolution to an analysis of the well known data on aptamer recognition of amino acids, in the hope of elucidating remnants of the primordial code. Here they make a very good observation that explains the superficially puzzling representation of both codons and anticodons in the aptamers selected for binding Arg, Ile and Tyr: the codons for these amino acids are self-complementary palindromes in positions 1-2 (eg, CGN for Arg), so it is not surprising that, as long as (say) the codon is over-represented in the aptamer, so will be the anticodon. This I believe solves a "pseudo-puzzle" in the aptamer data. They then continue with the discussion of the other type of palindromic relationship between codons and anticodons: codon positions 2-3 vs anticodon positions 1-2. The conclusion for Arg is that "*when for the same amino acid, arginine, we find in its RNA-binding sites a cognate, but not-CG-containing, triplet, it appears to be the anticodon, not codon". *And overall: *"Anticodons, not codons, are more often significantly over-represented in aa-binding sites of the RNA aptamers*". There is also another, more subtle pattern that comes out of the aptamer analysis: "*For amino acids encoded by dinucleotide-palindrome-containing triplets, their binding sites in RNA aptamers "prefer" the codon(1-2)/anticodon(2-3) motifs over the codon(2-3)/anticodon(1-2) ones in spite of their seemingly perfect symmetry*". The primary conclusion, of course, is that the 3^rd ^base of the over-represented triplet in aptamers is more important in aptamer-amino acid recognition, so the recognition is anticodon-based rather than codon-based. The meta-conclusion is that this recognition is a relic of the putative primordial, pre-translational code.

I am afraid I am rather skeptical of this entire line of reasoning - above all, because the aptamer data are weak, with the possible exception of only two amino acids, and these amino acids (Arg and Ile) are not the simplest ones or those that are generally thought to be primordial. I am just not convinced that the aptamer data are relevant at all. What is more, assuming there is some signal there, I am not sure it has anything to do with either codons and anticodons. The authors themselves are very emphatic on the statements that the primordial code had nothing to do with translation, and indeed, the classic results on specific aminoacylation of the CCA-arms (refs. [25,26]) do suggest the existence of a primordial operational code that might have had nothing to do with the present one.

The rest of the paper is a rather involved discussion of the potential relics of the pre-translation stages in the tRNA structures, in part, recapitulating the previous publications of the authors. The idea with the Fibonacci-like iterative process of tRNA evolution is of course very elegant and numerologically appealing but the entire scheme is quite speculative.

So does this paper report progress in our understanding of the origin and evolution of translation and the code? It is hard to give a 'yes' or 'no' answer. The paper ends with the overall conclusion that "*Translation without code does not make sense, but code without (and before) translation does". *I think this is good logic. I also applaud with the authors' approach of combining many different lines of evidence in approaching this formidable evolutionary puzzle. However, my overall position is that we still have not developed the right way to approach this problem, so all current scenarios are likely to be wrong in most details. In this situation, what matters is the quality of the discourse, and in this respect, the current paper will be helpful to any researcher interested in the problem.

#### Authors' Response

We have greatly appreciated the reviewer's comments and critique; our response is structured in the following three sections:

##### I. On the general relevance of aa-binding RNA aptamers data to the problem of the genetic code origin

Just like the singularity point in the origin and evolution of universe, the actual start of bilingual life (with nucleic acids, proteins and genetic code in between) was, is, and probably will always remain obscure. In such an "agnostic" (in Huxley's meaning) situation, one of the very few intellectual pursuits that a theoretician could afford to accept as not being hopelessly speculative would be to examine, by all conceivable means (including *in vitro *experiments with aa-specific RNA aptamers), the crucial "key-lock vs. frozen accident" alternative. Naturally, *in vitro *selection of RNA aptamers (aimed at an increasingly more specific recognition of a particular amino acid) has little, if anything, in common with shaping of the genetic code that actually took place in early evolution. Apparently, the real code developed stepwise following the classic scheme of gene duplications with subsequent specifications, regardless of whether it have started with a very specific stereo-chemical "key-lock", a true "frozen accident", or possibly something in between --- a "frozen *stereo-chemical *accident" ([16] see also [10]). In contrast, SELEX experiments of the Yarus type always start with *an absolutely random RNA sequence. *But if so, then the excesses one observes in selexed aa-binding RNA sites --- excess of cognate anticodons in general, and excess of anticodon (2-3)/codon(1-2) tetraplets in particular --- are all the more impressive.

##### II. More on excess of anticodons in aa-binding sites of RNA aptamers

Logically, if we accept that p-aaRSs must have been preceded by r-aaRSs, then we have to face the problem of interaction between a particular aa and its cognate r-aaRS, and this, in a nutshell, is the proverbial problem of the genetic code origin. Specifically, a whole cluster of interrelated questions arises, the most important (in our estimation) ones being: (1) was this interaction absolutely random, or stereo-chemically selective (at least weakly), and (2) did this interaction have anything in common with the real genetic coding?

The similarity of aa-binding sites in RNA aptamers independently selexed for a given amino acid casts doubt on the randomness. Moreover, the significant excess of cognate anticodons within these sites strengthens the hypothesis of underlying stereo-chemical preference. The RNA aptamer data are statistically compelling in this regard (see also the detailed analysis in [12]), and the question one might ask is rather ***how to account for this striking level of significance by any other means?***

Previously, one of us (ES) has formulated this precisely: logic dictates the hypothesis of direct interactions between RNA and amino acids in the emerging RNP life, but corresponding binding sites may not have anything to do with the current cognate triplets [9]. In this case, the codon/anticodon triplets would have been an early operational code of sorts (in a sense that they would have been symbolic/conventional, rather than iconic). Then, of course, we should be able to demonstrate some other sequences being specific binders. The experiments, first suggested in [90], are now known as Yarus type SELEX experiments; our analyses thereof have revealed anticodonic "predominance", and so we tentatively conclude that early coding was, so to speak, iconic and anticodonic.

Two of us (SNR and ASR), just like the Reviewer 1 (and for similar reasons), initially also made light of the stereo-chemical hypothesis until (1) revealing the sub-code for two modes of tRNA recognition by aaRSs (Figure 1B), and (2) noticing that the puzzling confusion-prone simultaneous presence of codons in aa-binding sites of selexed RNA aptamers might have been simply a hitch-hiking by-product of the anticodon-targeted selection (discussed in this paper). We feel that this observation is in fact more important than a mere resolution of the persistent but superficial puzzle --- and it certainly increases the significance of RNA aptamer data in the context of the problem of genetic code origin. In principle, the complementarity of codons and anticodons means that there is no difference whether it is the former or the latter that will prevail in aa-binding sites of RNAs. However, the fact that it is actually the latter (and we present not one but many independent pieces of evidence in support of the anticodon's prevalence) tells us a lot about the consistency of the primacy of anticodons (by origin) with logically necessary primacy of coding. As a matter of fact, it is mildly ironic that the aa-cognate triplets in mRNAs were named "codons" (ensuring their complements in tRNAs being named "anticodons"). Strictly speaking, it would make more sense to flip the terms --- if anticodons did emerge first, as the aa-specific triplets in ribozymes of the primordial RNA life (perhaps long before amino acids were recruited into translation), they deserve to be named "codons". Consequently, triplets in mRNAs would become "anticodons" (thus reflecting their origin as complementary copies of aa-specific triplets).

##### III. On primordial amino acids, operational code and sub-code for two modes of tRNA recognition by putative r-aaRSs

Aminoacylation of a tRNA molecule from two, major and minor grove, sides (Figure 1C) is supposed to be a very ancient feature of life. By complementary transformation of the genetic code table (Figure 1B), we have revealed its internal "yin-yang" pattern (a sub-code of sorts) that minimizes errors of tRNA recognition by putative r-aaRSs for complementarily encoded amino acids [19,36]. This sub-code implies primacy of anticodons in the genetic code origin, but for simplest ("primordial"?) amino acids such as Ala and Gly, recognition of their proto-tRNAs by putative r-aaRSs might have been more acceptor- than anticodon-targeted, i.e. associated with the ancient operational code rather than the classic one (see [20] for details of this seeming "paradox"). Importantly, we do stress that in actuality there is no "paradox" here, and that in general the primordial code most likely had little, if anything, to do with translation (but, of course, not with coding *per se)*. Quite the opposite, in a series of papers [18-20,35,36] we pursued the idea (and consolidated the data in support of showing) that (1) the primordial code was actually operational (and only operational), but nevertheless (2) these two codes - operational code of tRNA aminoacylation embodied mostly in the acceptor stem [25,26] and the classic code associated with anticodons by which cells are reading mRNAs during translation - might have had a common ribozymes-implemented ancestor [18-20,35,36]. If so, classic experimental results of proper specific aminoacylation of tRNAs truncated to mini- or even micro-helix [25,26] and *in vitro *selection of aa-specific RNA aptamers [12] complement rather than exclude each other --- which is one of the leitmotifs of the present paper.

As far as Ala, Gly or any other (presumably) primordial amino acids are concerned, at this time we are not aware of any reports stressing not the excess (or deficiency) of their cognate anticodons (codons) in aa-binding sites of RNA aptamers, but rather the successful selection of such RNA aptamers at all. Thus, it remains unclear whether such excess in fact exists; further, more refined, experimental attempts with selection of RNA aptamers for such amino acids would be most welcome.

### Reviewer's report 2

Wentao Ma, College of life sciences, Wuhan University, P.R. China, nominated by Juergen Brosius.

#### Reviewer Comments

The origin of genetic code and translation system is a problem (or two related problems) full of controversies. The reason is that the translation system is very complicated and the coding principle is not clear (e.g. not clear as the base-pairing mechanism evolved in the replication of DNA or RNA, or their interdependent synthesis). According to the Darwinian Continuity principle (i.e. "evolution has no foresight") [7], the emergence of the complicated system, including its components as well as the coding principle, should have included quite a few intermediate steps. This manuscript, following the idea of a previous hypothesis (CCH, Coding Coenzyme Handles [9]), emphasized (with new evidence) that the coding principle should have emerged before translation. This idea has its intrinsic feature to explain the origin of the coding principle considering that "evolution has no foresight". The evidence came mainly from a detailed analysis of the updated data in experiments of RNA aa-aptamers [12]. Overall, the argument is reasonable, but, in my opinion, some detailed deductions or assertions need a more cautiously examination.

Based on the analysis on aptamer data of three amino acids containing both their anticodons and codons, the authors asserted reasonably that such cases should be associated with the palindrome-dinucleotides. Together with data of other amino acids in [12] and also recent evidence in [31,33], it is also reasonable to conclude that anticodons, not codons, are more often significantly over-represented in aa-binding sites, and that in some cases, "codons might simply follow anticodons (like hitch hikers)" This conclusion is "welcome" by the stereochemical theory on the origin of the genetic code, considering the previous statement, "both anticodons and codons are over-represented in aa-binding sites", is ambiguous and hard to explain [7].

"For amino acids encoded by dinucleotide-palindrome-containing triplets, their binding sites in RNA aptamers "prefer" the codon(1-2)/anticodon(2-3) motifs over the codon(2-3)/anticodon(1-2) ones in spite of their seemingly perfect symmetry." This conclusion seems also to be supported by the data in [12]. Indeed, the situation could be interpreted as the 1-2 nucleotides of the codon contribute more to the specificity of interaction, or the 2-3 nucleotides of the anticodon contribute more to the specificity of interaction. Then, the latter interpretation could be accepted considering the above conclusion that it might be anticodons that actually associated with cognate amino acids. Thus, a new, interesting assertion, stronger than that in the CCH hypothesis, could be phrased as "the degeneracy of 1st position in anticodons (3rd position in 'future' codons) also appears to have originated before translation". In this context, it is also attractive to attribute "the fact that codon's 3rd nt is more degenerated than the anticodon's 1st nt" to the cause that anticodons should have emerged before codons, which would be used only in the "future" translation system.

"The motif 5'-CGCG-3' is of a special interest (Figure 2), because one can see it in two ways: CGC is a codon(1-2) for CG palindrome and, simultaneously, it is also a codon(2-3) for GC palindrome. The overlapping GCG anticodon, in its turn, can be considered as the (2-3) or (1-2) one for CG and GC, respectively."

I think that this description has some problems. The corresponding motif should be 5'-GCGC-3' when CGC is a codon(1-2) and GCG an anticodon(2-3) for CG palindrome, while the corresponding motif should be 5'-CGCG-3' when CGC is a codon(2-3) and GCG an anticodon(1-2) for GC palindrome.

"This ambiguity is fraught with confusion for coding. Very telling, therefore, is the fact that Arg-binding sites do not contain the 5'-CGCG-3' motif at all, in contrast to the above three motifs of the codon(1-2)/anticodon(2-3) type. This fact becomes even more telling if we take in account presence of 5'-CGCG-3' beyond aa-binding sites ([12]: see, in the Arg list, cases 17, F2e, F2f, and F2U)".

Now that aptamers have nothing to do with translation, how comes the ambiguity for coding? The logic is hard to understand. The true one might be that the 5'-CGCG-3' motif is a representation of codon(2-3)/anticodon(1-2), thus not appearing. If so, a directly contrary motif should be 5'-GCGC-3' (as I mentioned above), which should be abundant. However, the analysis or comment on 5'-GCGC-3' does not appear in the manuscript.

#### Authors' Response

We found the reviewer's comments and critique particularly incisive; the two major issues are discussed below.

First of all, one should take into account that for arginine these tetraplets, 5'-CGCG-3' and 5'-GCGC-3', are both codon(2-3)/anticodon(1-2) relative to the GC palindrome and, simultaneously, are both codon(1-2)/anticodon(2-3) relative to the CG palindrome, the only difference being the dinucleotide with which anticodon GCG and codon CGC overlap --- GC in the 5'-CGCG-3' case, and CG in the 5'-GCGC-3' case. Accordingly, if anticodon GCG is a driver, and GC palindrome is under consideration, then the corresponding tetraplet containing Arg's codon is 5'-CGCG-3' (and only 5'-CGCG-3'). On the contrary, if anticodon GCG is again a driver, but it is CG palindrome that is under consideration, then the corresponding tetraplets are 5'-GCGN-3', *including *5'-GCGC-3'.

Importantly, it is the CG at 1-2 positions of codon (2-3 positions of anticodon) that actually specifies Arg, whereas the GC at 2-3 positions of codon (1-2 positions of anticodon) is irrelevant (specifying Ala). It thus appears that codon CGC might have "formally" accompanied (as a hitch-hiker) anticodon GCG in 5'-CGCG-3' via the Arg-unrelated GC dinucleotide, and that is precisely why we chose to *deliberately *focus our description on 5'-CGCG-3'. The result - absence of this motif in Arg-binding sites - speaks for itself. Remarkably, in independently selexed Ile-binding sites we also do not see (and probably exactly for the same reason) the 5'-AUAU-3' tetraplet in which anticodon UAU supposedly drives codon AUA as a hitch-hiker but via the central Ile-irrelevant UA palindrome (in the manuscript, we stress this fact just after describing the arginine case).

**Figure 9:**
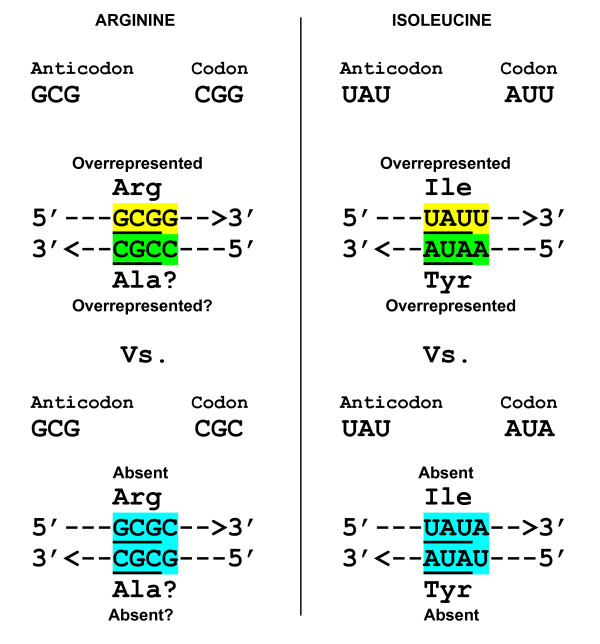
**Comparison of overrepresented and absent coding tetraplets in Arg-, Ile- and Tyr-binding sites of "selexed" RNA aptamers**. The 3'-CGCC-5' is questionable as a possible coding motif for putative Ala-binding site. Anticodons are underlined. Complementary "yellow" and "green" tetraplets cannot be confused (in contrast to self-complementary "blue" ones).

Needless to say, from the very beginning we have checked all possible Arg-related tetraplets including those, in which anticodon GCG was supposed to be a driver. The results were (1) excess of 5'-GCGG-3' and (2) *complete absence *of all others, including the 5'-GCGC-3'. Again, one can see exactly the same pattern in Ile-binding sites with anticodon UAU as a driver: a great excess of the 5'-UAUU-3' and complete absence of all others, including the 5'-UAUA-3' (Figure 9).

Furthermore, the UAU anticodon of Ile is a codon for Tyr. However, the latter is not significantly overrepresented in Tyr-specific sites of RNA aptamers. Symmetrically, AUA is a significantly overrepresented triplet, as an anticodon, in Tyr-binding sites but not, as a codon, in Ile-binding ones. Thus, it appears that *coding triplets in Ile-binding sites preclude confusion with Tyr (and *vice versa*) as if the RNAs selected for direct affinity to a specific amino acid have already been protected from confusion with its complementarily encoded partner. *This is surprising, because the RNA aptamers have not been specifically selected to avoid non-cognate amino acids. We have decided to address this issue in a more nuanced fashion elsewhere (Szathmáry and Rodin, in preparation); however, the reviewer's remarks led us to restoring the original paragraph of the manuscript here, in the present discussion.

Tyr-binding sites show overrepresentation of the characteristic 5'-RAUA-3' motifs (with AUA anticodon): 5'-GAUA-3' and 5'-AAUA-3', which is a precise complement of the characteristic 5'-UAUU-3' motif (with UAU anticodon) that happens to be overrepresented in Ile-binding sites (see Figure 3 and [12]). This fact is consistent with our hypothesis of two earliest r-aaRS precursors that have been complementary to each other [18-20]. Importantly, *these coding tetraplets, 5'-UAUU-3' and 5'-AAUA-3', cannot be confused*. In contrast, coding tetraplets (in putative r-aaRSs) formed by complementary anticodon and codon, are indeed confusion-prone simply because any such tetraplet is a perfect self-complementary palindrome. For example, Ile's anticodon UAU and its complement AUA form the 5'-UAUA-3' palindrome, the complementary image of which is, again, 5'-UAUA-3'. Same holds for tyrosine as well as complementarily encoded arginine and alanine. In such cases, the two complementary r-aaRSs might have had virtually the same aa-coding sites, leading to the high risk of wrong aminoacylation --- accordingly, they would have been selected against.

Obviously, the correct logical flow should be in reverse: selection for stereo-affinity of a given amino acid (for example, Ile) to the oligonucleotides containing this aa-cognate anticodon UAU within the 5'-UAUU-3' tetramer automatically entails selection for the presence of AUA within 5'-AAUA-3' in the complementary sequence which happens to be preferred by Tyr - the Ile's complementary partner *in the real genetic code*. Of course, it is unlikely that the actual shaping of the genetic code had much in common with this *in vitro *multi-step selection of RNA aptamers each time beginning with a random sequence (see our response to reviewer 1). Therefore, the detection of these mutually complementary and "non-confusable" tetraplets, 5'-UAUU-3' and 5'-AAUA-3' (and, on the contrary, complete absence of "confusable" 5'-UAUA-3' and 5'-AUAU-3') within Ile- and Tyr-binding sites of *independently selected *RNAs is all the more meaningful. At this time, we do not suggest any mechanistic explanation for this somewhat surprising result but it shows us that there might have been, indeed, certain structural prerequisites for two complementary modes of tRNA recognition by aaRSs, firstly ribozymes then enzymes. Moreover, this result indirectly supports, in our opinion, two basic hypotheses: (1) that primary stereo-affinity did play an essential role in the origin of the genetic code, and (2) that *even before translation *amino acids might have been engaged in coding by complementary pairs rather than one by one. The above immediately suggests a relatively simple idea for the selex experiment control - to use both plus and minus RNA sequences in selection of aa-binding RNA aptamers, so that selection for one aa-binding site would facilitate starting conditions for selection of its complementary partners.

To our knowledge, there were no reports of successful selection of RNA aptamers for Ala, the complementary partner of Arg (for possible reasons see [20], the main text and our response to Reviewer 1), whereas the available data on Ile- and Tyr-binding sites are consistent with this hypothesis. Moreover, as one can see in Figure 9, by substituting C for A and G for U, we move (1) from the Arg-coding CG palindrome-dinucleotide to the Ile-coding AU palindrome-dinucleotide, (2) from the 5'-GCGG-3' motif, overrepresented in Arg-binding sites, to the 5'-UAUU-3' motif, overrepresented in Ile-binding sites, and (3) from 5'-GCGC-3', which is completely absent in Arg-binding sites to 5'-UAUA-3', which is completely absent in Ile-binding sites. The same result can be obtained by moving from Ile to Arg, i.e. by replacing A for C and U for G. We believe that this striking parallelism between coding motifs of such different amino acids as Arg and Ile sends us an important message about the origins of the genetic code. In particular, if at some point in the future the Yarus-type experiments "selexed" for alanine prove to be successful, we would not be particularly surprised by finding the 5'-CCGC-3' motif (with anticodon CGC) in Ala-binding sites of RNA aptamers.

Finally, we must confess that, in turn, we do not quite understand the reviewer's perplexity expressed in "Now that aptamers have nothing to do with translation, how comes the ambiguity for coding?" The gist of our report is that the origin of the coding system does not necessarily have to be tied to translation. Furthermore, as coding preceded translation, we do not need (and thus avoid being caught in the implicit trap of) the foresight evolution. And, the risk of ambiguous anticodon-to-aa assignment, while originally of crucial importance for primordial coding in the RNA world, was of less, if any, relevance for translation which most likely evolved later. In the RNA world, errors of RNA aminoacylation might have been serious enough to be selected against. In conclusion, this remains a high priority task on our agenda - to find out which component of the genetic coding ambiguity has been minimized before (and outside of), and which --- after (and in co-evolution with) the development of translation machinery.

#### Reviewer Comments

"Literally: 3 + 4 = 7, 4 + 7 = 11, 7 + 11 = 18, 11 + 18 = 29, 18 + 29 = 47, and finally 29 + 47 = 76!" This seems to be a coincidence. The Fibonacci-like iterative process in the model of tRNA growth (Figure 5) is not based on an explanation of structure-function relationship. For example, why tRNAs should grow in such a way? or, what is the driving force for the growth? Without a detailed explanation on these intermediate steps, the model is not presented in a way consistent with the Darwinian Continuity principle.

Actually, there are quite a lot other interpretations based on the authors' previous work that seems to be problematic because there is not a consideration on those intermediates steps involve in the origin of translation system, for example, those concerning r-aaRSs and the operational code. The r-aaRSs ("implementers") should be a set of functional RNA molecules in addition to the set of tRNAs ("adaptors"). If they should have existed, what is the original source of these RNA molecules before they were recruited into the translation system? In addition, what should their structure be like to implement their function? Similarly, those interpretations concerning the operational code are also obscure. If the operational code should indeed have worked in the RNA world, and have occurred before the emergence of the anticodon loop, what advantage would drive the later emergence of the anticodon loop? On these points, I do not mean that the events involved is impossible, instead, I tend to agree the view of Koonin and Novozhilov ("Origin and evolution of the genetic code: the univeral enigma" Iubmb Life 2009, 61: 99-111), namely, "a real understanding of the code origin and evolution is likely to be attainable only in conjunction with a credible scenario for the evolution of the coding principle itself and the translation system". The extended discussion in the present manuscript on these events seems to give readers an impression that there are too many assumptions without a detailed interpretation solidly based on a scenario according to the Darwinian Continuity principle. Perhaps a better choice of the manuscript is to focus its discussion on its formal schemes mentioned above, and extend the discussion only a little to the issues concerning the authors' previously work on r-aaRSs, the operational code and others.

#### Authors' Response

We are certainly aware of the translation problem, and we are not (at this time, anyway) staking a claim to the full, comprehensive solution --- rather, we suggest a number of clues to that effect.

As far as possible intermediate advantages in evolution of the operational code, putative r-aaRSs, and tRNAs are concerned, we feel that the reviewer might, figuratively speaking, be asking too much --- and not just from the proposed scenario, but from the field of the genetic code origin research in general. Moving on to the specifics of the Fibonacci-like iteration coincidence, we would like to touch briefly on the following two aspects:

First, the internal periodicity of tRNA sequences and certain other considerations suggest to many investigators that tRNA precursors were at first much shorter (why would they be longer?), but have grown afterwards. If so, the following important questions arise: (1) how is it possible to achieve the same structure in the simultaneously growing molecules? and, (2) how could this growth reflect the principle of evolutionary continuity, where the next stage inherits useful functions gained at the previous stage? For (1), we show how this could be perfectly possible, and for (2) we demonstrate that the process of Fibonacci-like iterative growth fits the continuity principle (at least outwardly). To tell the truth, it was not the yet another example of the golden ratio implementation in Nature that held particular appeal to us, but rather the idea of the regularized and coordinated growth of the initial coding tri- and tetra-nucleotides "towards" one and the same final cloverleaf, a process appealingly consistent with the continuity principle.

Second, when studying evolution *ab simplecioribus ad complexiora*, it goes without saying that, in order to make any stepwise evolutionary scenario workable, one should think about "Darwinian" motivation for each step. Gradual shaping of code adaptors into the final tRNA cloverleaf is no exception; however, we can only guess which specific agents in the "late" RNA world (with translation already emerging) could drive this process. This said, the tRNA molecule is truly a molecule "for all seasons" [91] --- it could have had many opportunities to do so. We consider this issue worthy of comprehensive treatment, and it will be discussed elsewhere (Szathmáry and Rodin, in preparation).

### Reviewer's report 3

Anthony Poole, Stockholm University, Stockholm, Sweden

#### Reviewer Comments

This paper is a wonderful piece of detective work that at the same time synthesizes many separate observations and theories on the origin of the genetic code and tRNAs. I really found nothing in here that requires substantial comment or clarification, other than to say it is a thoroughly interesting read - real food for thought. The analysis of results of amino acid binding sites in vitro selected RNA aptamers is thorough, and the Fibonacci process-inspired model for the evolution of tRNAs is truly insightful and thought-provoking. Perhaps one of the most interesting points of this process is that it yields a very precise stepwise model wherein tRNAs can converge independently from unrelated short RNA aptamers that bind amino acids upon a common length (and structure), ultimately containing both operational and genetic codes.

#### Authors' Response

We are very grateful to Reviewer 3 for this encouraging, succinct and yet precise formulation of what we have actually set out to achieve when writing the manuscript.
